# Dna is a New Target of Parp3

**DOI:** 10.1038/s41598-018-22673-3

**Published:** 2018-03-08

**Authors:** E. A. Belousova, А. A. Ishchenko, O. I. Lavrik

**Affiliations:** 1 Institute of Chemical Biology and Fundamental Medicine (ICBFM), SB RAS, Lavrentiev Av. 8, Novosibirsk, 630090 Russia; 2 Groupe Réparation de l’ADN, Equipe Labellisée par la Ligue Nationale Contre le Cancer, CNRS UMR8200, Univ. Paris-Sud, Université Paris-Saclay, F-94805 Villejuif, France; 3 Gustave Roussy, Université Paris-Saclay, F-94805 Villejuif, France; 4 Novosibirsk State University, Pirogov Str. 2, Novosibirsk, 630090 Russia

## Abstract

Most members of the poly(ADP-ribose)polymerase family, PARP family, have a catalytic activity that involves the transfer of ADP-ribose from a beta-NAD+-molecule to protein acceptors. It was recently discovered by Talhaoui *et al*. that DNA-dependent PARP1 and PARP2 can also modify DNA. Here, we demonstrate that DNA-dependent PARP3 can modify DNA and form a specific primed structure for further use by the repair proteins. We demonstrated that gapped DNA that was ADP-ribosylated by PARP3 could be ligated to double-stranded DNA by DNA ligases. Moreover, this ADP-ribosylated DNA could serve as a primed DNA substrate for PAR chain elongation by the purified proteins PARP1 and PARP2 as well as by cell-free extracts. We suggest that this ADP-ribose modification can be involved in cellular pathways that are important for cell survival in the process of double-strand break formation.

## Introduction

Poly(ADP-ribose)polymerases, PARPs, represent a protein family that is involved in a number of crucial cellular processes that are linked to genomic DNA integrity such as DNA repair, genome stability, and cellular stress responses^1^. The entire human family includes 17 members with very different structures and cellular functions but that are related by the presence of the PARP signature, a conserved PARP catalytic domain^2^. Most members of the family perform the catalytic activity of transferring ADP-ribose from the beta-NAD^+^-molecule to the acceptors; however, only three of them, PARP1, PARP2 and PARP3, possess DNA-dependent (ADP-ribose)transferase activity^1^. Interestingly, PARP1 and PARP2 catalyse the synthesis of a long stretch of poly(ADP-ribose)polymers, whereas some results have indicated that PARP3 is a mono(ADP-ribose)transferase^3^. Since the discovery of the first PARP reaction in 1963, only proteins have been viewed as exclusive acceptors for the PARP enzymes^4^. A large number of protein acceptors have been found for the most investigated member, PARP1, and for other active members^3,5,6^. This means that PARPs can potentially catalyse the reactions that utilize a large range of acceptors that differ in their chemical nature. However, as shown recently, the spectrum of PARP acceptors is not confined to amino acids. Indeed, poly(ADP-ribose)polymerases 1 and 2 can catalyse the synthesis of a poly(ADP-ribose) attached to DNA^7^.

Since PARP3, like PARP1 and PARP2, is a DNA-dependent family member, it was interesting to examine the capacity of recombinant human PARP3 to modify DNA. The principal features of the DNA modification, as well as a detailed characterisation of the DNA substrate preferences for PARP3 and PARP2, have been provided in another study that was recently published^8^. Here, to further expand our knowledge about this PARP3 capacity, we used partial DNA duplexes to study the action of PARP3 and investigate the detailed the nature of the DNA modification.

## Results

### Activity of human PARP3 on partial DNA duplexes

**Figure 1:**
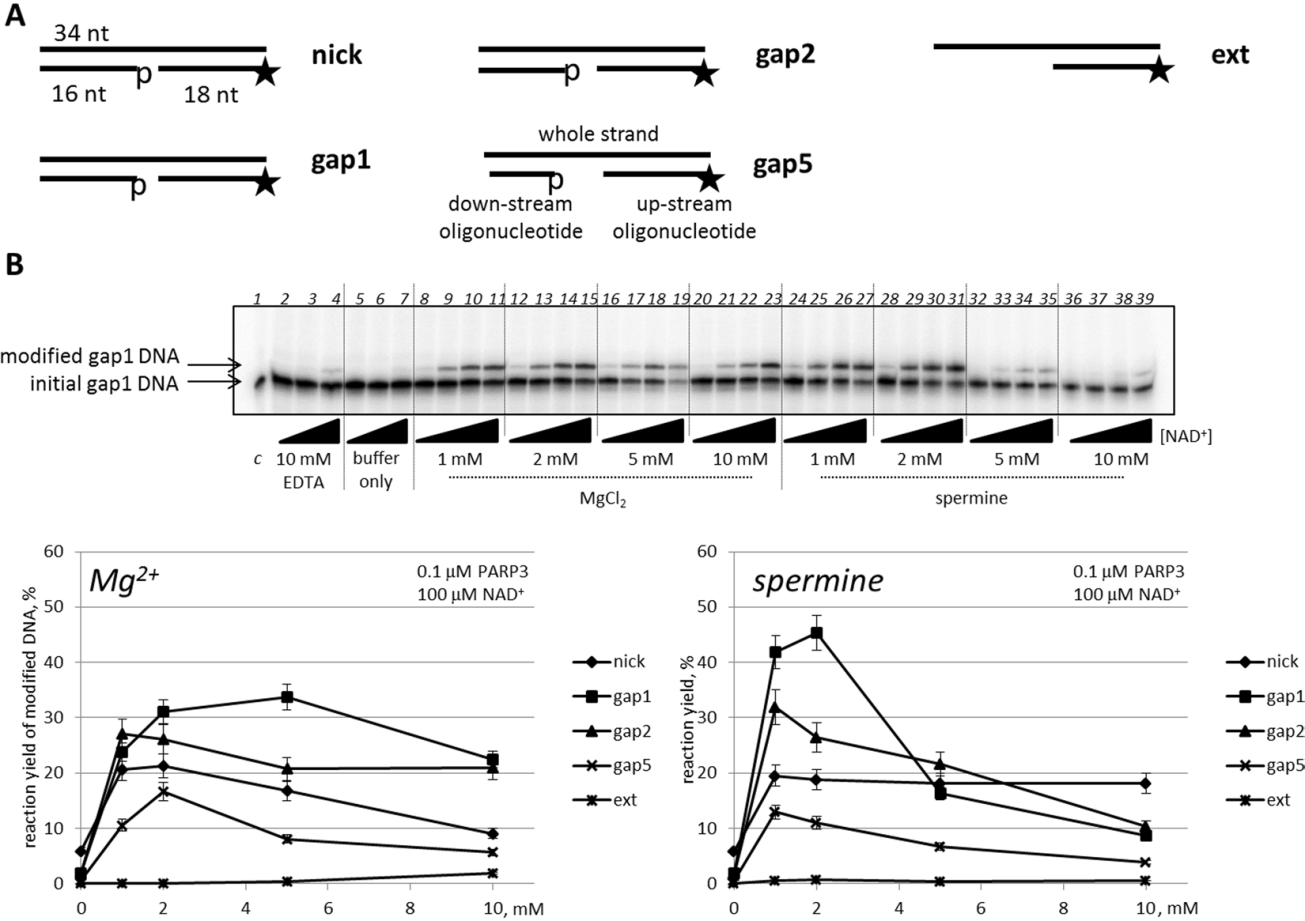
Basic activity of PARP3 in DNA ADP-ribosylation. (**A**) Schematic representation of the DNA duplexes used. The asterisk marks the [^32^P]-label. (**B**) Activity of 0.1 µM PARP3 on gap1 in the absence (lanes 5–7) or presence of 10 mM EDTA (lanes 2–4), 1, 2, 5 and 10 mM MgCl_2_ (lanes 8–23) or 1, 2, 5 and 10 mM spermine (lanes 24–39). The reactions were performed using increasing concentration of NAD^+^ (1, 10, 100 and 1000 µM). (**C**) The reaction yield of modified DNA using various initial DNA substrates catalysed by 0.1 µM PARP3 with 100 µM NAD^+^.

Here, we tested the capacity of recombinant human PARP3 to modify partial DNA duplexes according to the newly discovered DNA-modification activity of PARP1 and PARP2^7^. For this, we decided to use partial DNA duplexes containing a few nucleotide gaps with 5′-phosphates on both the up- and downstream oligonucleotides as the most prominent DNA substrate with which to demonstrate the PARP1 DNA-modification activity (Fig. 1A). A DNA substrate with a protruding 5′-end was also used. Because the DNA-dependent PARP activity for the synthesis of poly(ADP-ribose) relies on various co-factors, we provided evaluated the presence of magnesium ions, spermine or EDTA in the experiment (Fig. 1B). We varied the concentrations of the co-factors and the NAD^+^-substrate as well as the protein concentration in the presence of various DNA substrates to elucidate the principal features and optimal conditions of PARP3 activity. The results showed that PARP3 can produce up to 55% modified DNA in the presence of the co-factors and even up to 10% modified DNA in the presence of 10 mM EDTA (data with EDTA not shown). Since PARP3 generates mono(APD-ribose)-modified proteins, a similar modification of DNA by the enzyme was anticipated. As expected, we observed a slight decrease in the mobility of modified [^32^P]-labelled DNA compared to the initial DNA mobility during electrophoresis. Based on the obtained results, we chose 0.1 µM PARP3 as the optimal concentration for further investigations, for which the co-factors and NAD^+^-substrate concentrations had been titrated to determine the overall optimal reaction conditions. The final reaction conditions included 0.02 µM DNA substrate, 0.1 µM PARP3, 2 mM MgCl_2_ or 2 mM spermine and a concentration range of NAD^+^ for all DNA substrates. The highest level of modification was observed with one-window gapped DNA. Thus, we chose this substrate for further investigations.

### The nature of the modification

**Figure 2:**
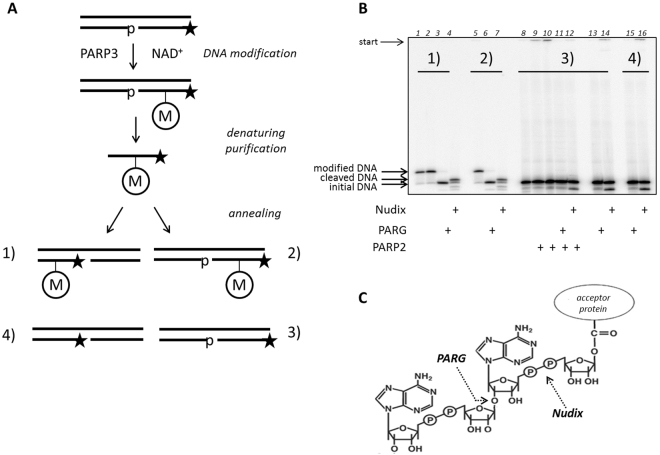
The cleavage of modified DNA by the activity of the glycohydrolases Nudix and PARG. (**A**) Schematic diagram of the preparation of PARP3 modified and unmodified DNA substrates. “M” inside the circle marks the modification induced by PARP3. The asterisk marks the [^32^P]-label. (**B**) Degradation of the different DNAs by Nudix and PARG (for details see Materials and Methods). Lane 1 – modified DNA1, lane 2 – modified DNA1 after 20 h incubation at 37 °C, lane 3 – modified DNA1 cleaved by PARG, lane 4 - modified DNA1 cleaved by Nudix, lane 5 – modified DNA2 after 20 h incubation at 37 °C, lane 6 – modified DNA1 cleaved by PARG, lane 7 - modified DNA1 cleaved by Nudix, lane 8 – DNA3, lane 9 –DNA3 modified by PARP2 after 30 min incubation at 37 °C, lane 10 - DNA3 modified by PARP2 after 20 h incubation at 37 °C, lane 11, 13 and 15 – DNAs treated by PARG, lane 12, 14 and 16 – DNAs treated by Nudix. (**C**) Schematic representation of the PAR-chain cleavage by PARG and Nudix.

To investigate the nature of the DNA modification carried out by PARP3, we first synthesised and purified preparative amounts of the modified [^32^P]-labelled DNA strand and constructed various modified DNA duplexes (Fig. 2A). According to the published data PARP3 is a mono(ADP-ribose)polymerase; thus, we expected that the same modification of DNA would be produced by the enzyme. To find circumstantial evidence in support of the fact that the modification was an ADP-ribose, we addressed the specific activity of the enzymes PARG and NUDT16, which are used by eukaryotic cells for PAR degradation. The first enzyme is the specific poly(ADP-ribose) glycohydrolase, which acts both as an endo- and exoglycosidase that catalyses the hydrolysis of poly(ADP-ribose) at the glycosidic (1′′–2′) linkage of the ribose-ribose bond to produce free ADP-ribose [reviewed in ref.^9^]. The second enzyme belongs to the NUDIX hydrolase superfamily, which can cleave the pyrophosphate bond even in the case of a single ADP-ribose unit^10^. Thus, using these two hydrolases, we obtained the enzymatic set to cleave the specific chemical PAR bonds: (1′′–2′) glycosidic and pyrophosphate. To identify the activity of PARG and Nudix on ADP-ribosylated DNA substrates we utilised the PARP2 modified PAR-DNA in the same experimental conditions (Fig. 2B., lanes 8–12). As a control for the DNA stability under these enzyme actions, we performed an additional experiment with unmodified DNA (Fig. 2B, lanes 13–16). The analysis of enzymatic cleavage of the various types of DNA duplexes revealed evidence that the modification is ADP-ribose (Fig. 2B).

**Figure 3:**
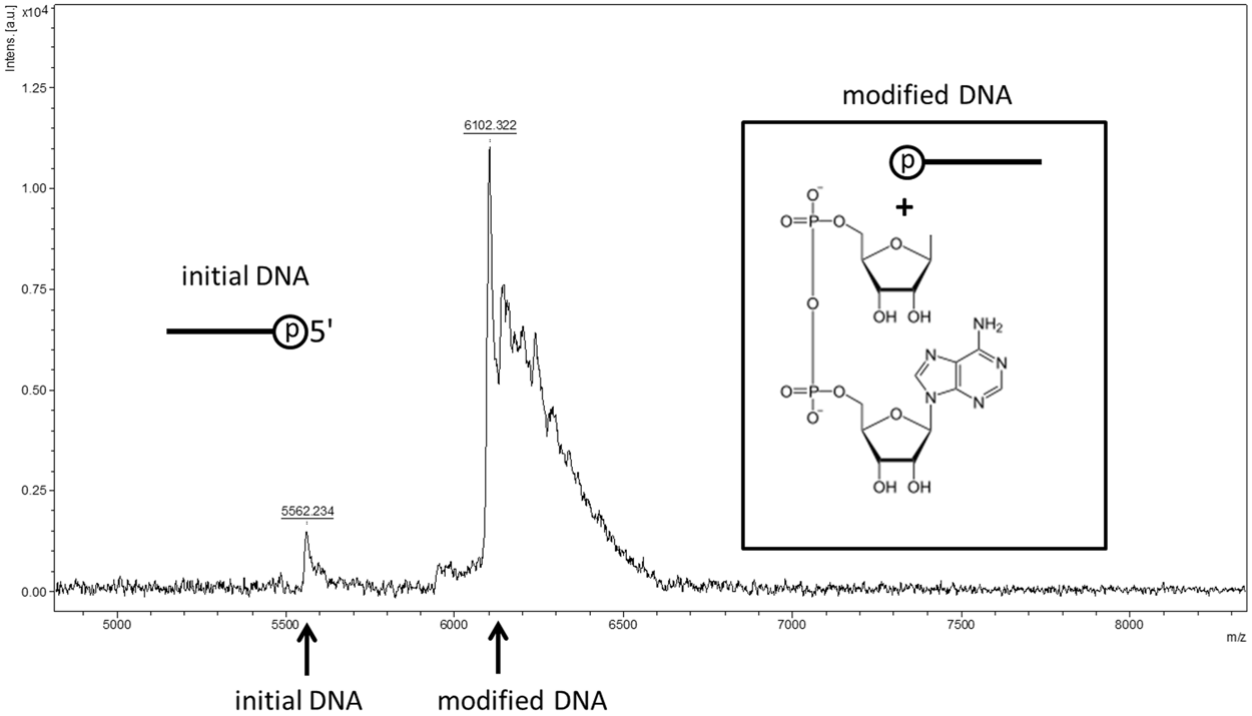
MALDI-TOF analysis of the purified modified oligonucleotide.

MALDI-TOF analysis of the modified oligonucleotide displayed the presence of two molecules with approximate molecular masses 6102 g/mol and 5562 g/mol, which is in agreement with the autoradiogram data (Fig. 3). The difference between these masses corresponds to the relative molecular mass of ADP-ribose. Therefore, based on the experimental data we can maintain that the modification carried by PARP3 is an ADP-ribose.

### The definition of the PARP3 acceptor

**Figure 4:**
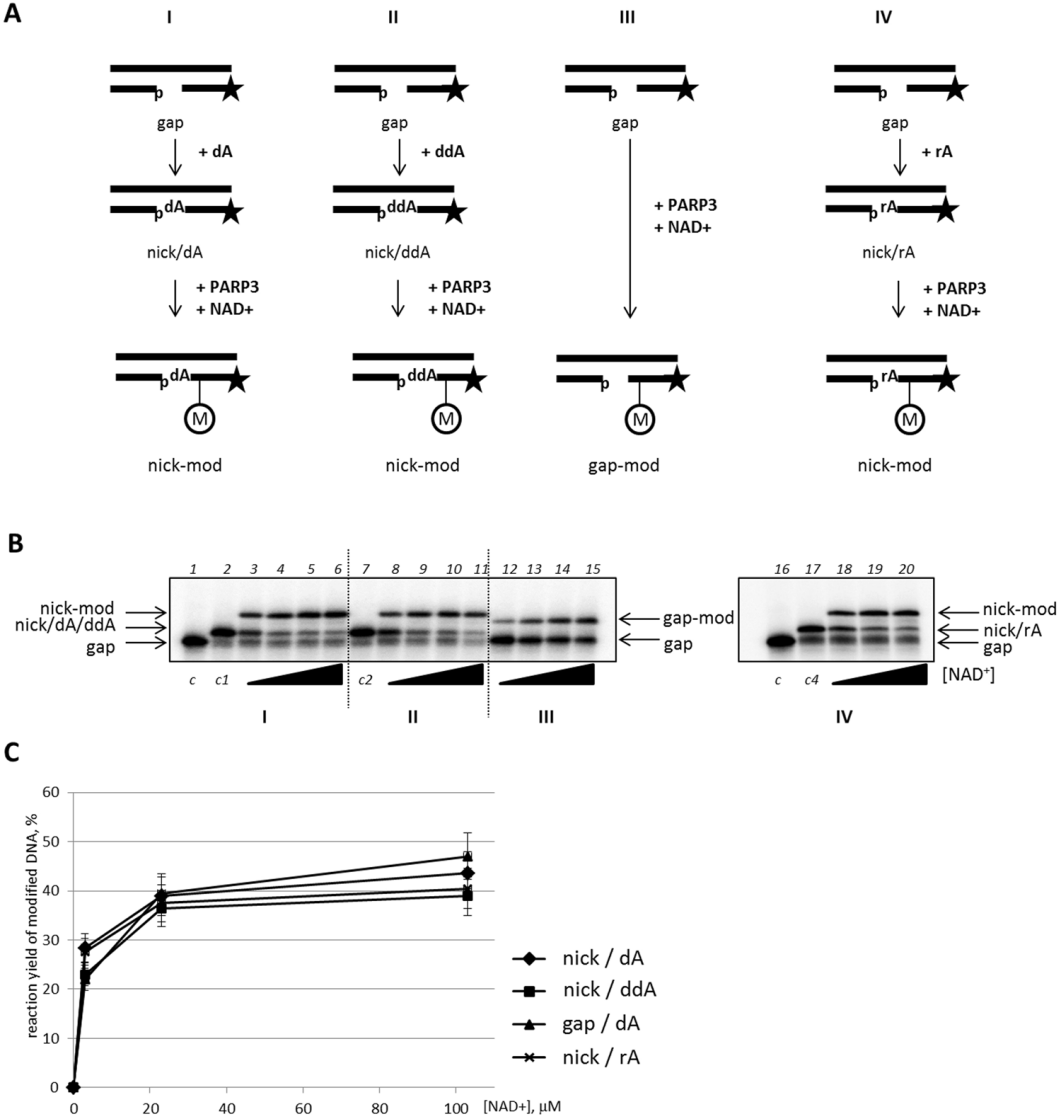
The influence of a 3′-end motif of the primer on PARP3 activity. (**A**) Schematic representation of the DNA transformation under full range reaction cycle. “M” inside the circle marks the ADP-ribose residue. The asterisk marks the [^32^P]-label. (**B**) One-window gapped DNA (lane 1, *c*) was used as a substrate to construct nicked DNA with dAMP (lane 2, *c1*), ddAMP (lane 7, *c2*) or rAMP (lane 17, *c4*) *in situ*. The activity of PARP3 on the DNA modification was investigated in the presence of 3, 20, 100 or 500 µM NAD^+^ (indicated by black triangles). (**C**) Quantitative analysis of the DNA PARP3 modification expressed as a percentage of the ADP-ribosylated DNA.

The modification carried out by PARP3 can be targeted at the various acceptor(s) in the DNA molecule. Basically, a 3′- or 5′-end position of the 5′-[^32^P]-labelled oligonucleotide could potentially serve as an active group. In the case of a 3′-end position of the oligonucleotide, this question can be comparatively easily resolved by variation of the 3′-nucleotide type. For this, we performed *in situ* synthesis using dATP, ddATP or ATP to provide obtained DNAs as substrates for the action of PARP3 (Fig. 4). Thus, DNA duplexes that were used as the substrates for PARP3 contained a 5′-[^32^P]-labelled priming oligonucleotide with a 3′-dAMP-, 3′-ddAMP- or 3′-AMP-end inside a single-stranded DNA break. It was found that the yield of the reaction products carried out by PARP3 did not distinguish among the various samples (Fig. 4B). This mean that the 3′-end did not undergo modification and that the modification performed is not in a nucleotide residue. Therefore, the important part is the 5′-group, which can be a phosphate or hydroxyl group or both.

**Figure 5:**
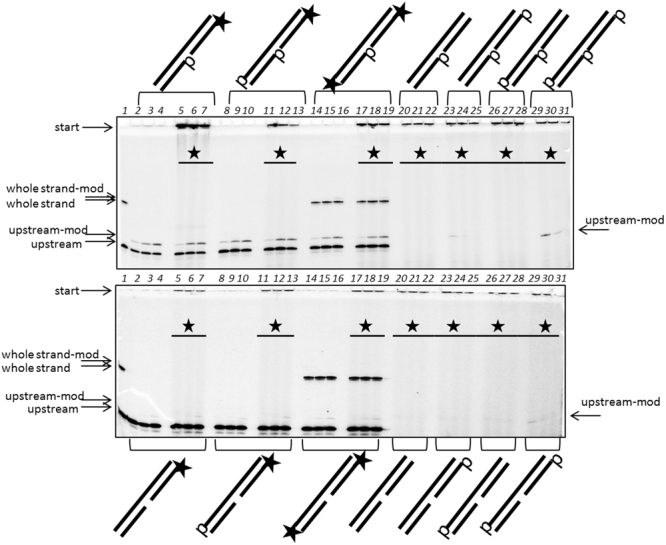
The influence of a 5′-phosphate or 5′-hydroxyl group in the different DNA strands on PARP3 activity. [^32^P] incorporation into DNA (lanes 2–19 at each panel and as indicated on the schematic view of DNA duplexes) or into the NAD^+^-molecule marked by asterisks (lanes 5–7, 11–13, 17–31 at each panel and as indicated on the gel areas). The reactions were performed under increasing concentrations of non-radioactive NAD^+^ (5, 50 or 500 µM NAD^+^). Lane 1 – initial electrophoretic mobility of the upstream and whole strand oligonucleotides.

To answer this question, we constructed various types of one-nucleotide gap DNA duplexes containing one-, two- or three phosphate/hydroxyl groups and tested the PARP3 activity on these substrates (Fig. 5). A radioactive label was introduced either into the 5′-end of the oligonucleotide or into the NAD^+^. The results obtained led us to the following conclusions. The modification can be primarily generated on the upstream oligonucleotide and occasionally on the whole strand of the DNA substrate. The 5′-phosphate group of the upstream oligonucleotide is required for the observation of DNA-modified PARP3 activity (compare in pairs lanes 20–22 and 23–25, 26–28 and 29–31 in the upper panel Fig. 5). The existence of the 5′-phosphate group on the downstream oligonucleotide led to a few-fold increase in the reaction yield (compare in pairs lanes 2–4 in the upper panel and lanes 2–4 in the lower panel, Fig. 5). Moreover, the 5′-phosphate group on the whole oligonucleotide promoted the accumulation of modified DNA (compare in pairs lanes 23–25 and 29–31 in the lower panel, Fig. 5). Based on these experimental results, we therefore, suggest that independent of its location the 5′-phosphate group is a DNA nucleophilic acceptor for the reaction catalysed by PARP3.

### Ligation assay

**Figure 6:**
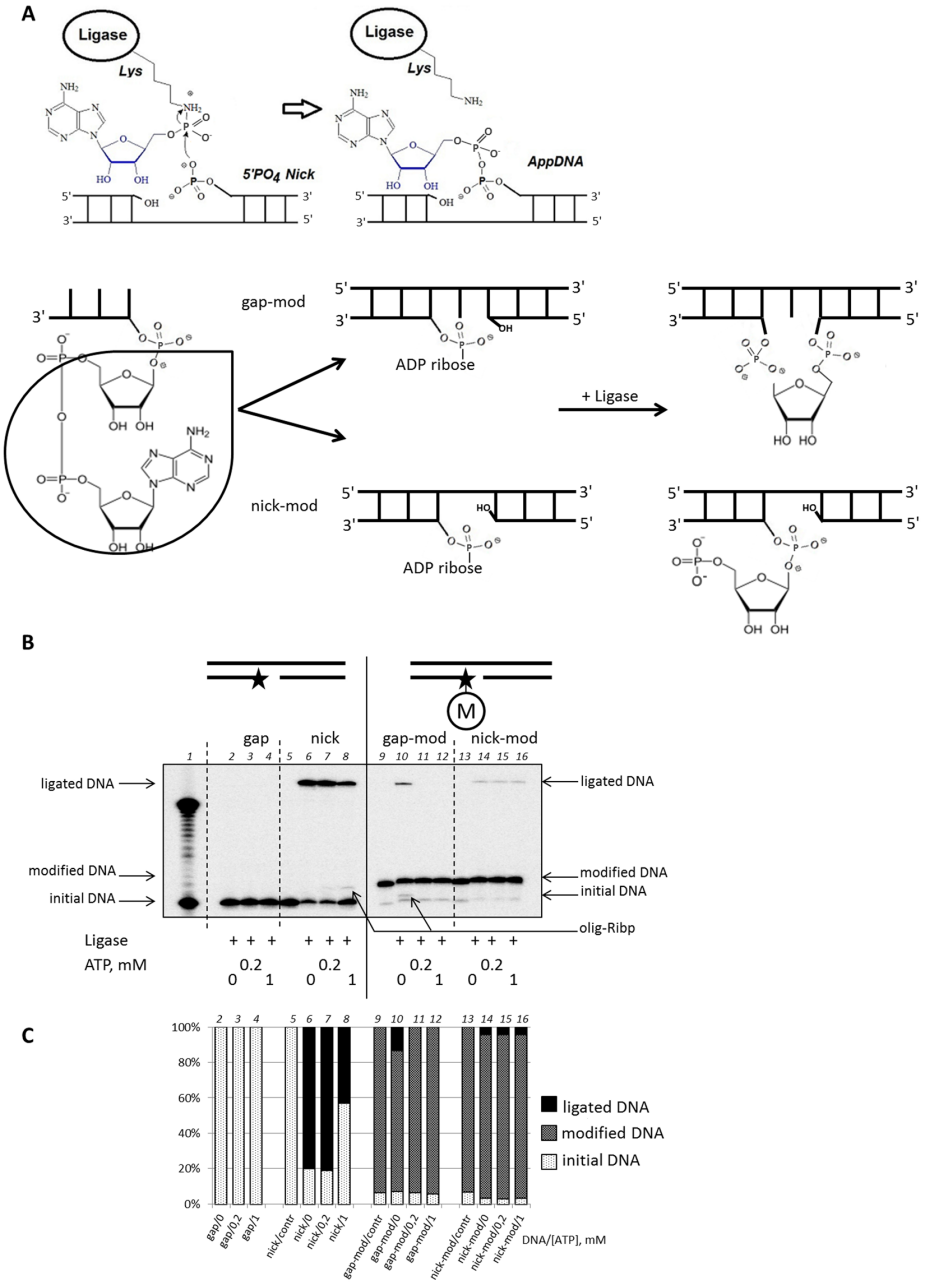
The ligation assay. (**A**) Schematic diagram depicting the mechanism for formation of 5′-AMP-DNA adducts under ligation (upper panel) and the set of products under ligation of various ADP-ribosylated DNA substrates (bottom panel). (**B**) Ligation of the one-window gapped (lines 2–4 and 10–12) or nicked DNA substrates (lanes 6–8 and 14–16) by DNA ligase IIIa. Lane 1 shows the DNA electrophoretic mobility from 18 to 34 nt; lanes 5, 9 and 13 show the initial mobility of [^32^P]-labelled DNA strand. Substrates gap-mod, gap and nick-mod, and nick represent one-window gapped or nicked DNA annealed with the various whole DNA strands and containing or not the ADP-ribose residue at the 5′-end of the [^32^P]-labelled oligonucleotide, respectively. “M” inside the circle marks the modification induced by PARP3. The asterisk marks the [^32^P]-label. (**C**) Quantitative analysis of the ligation assay reactions. The bars in the charts correspond to the respective lanes on the upper autoradiogram.

It is known that in the absence of a nucleic acid substrate, ligases including the NAD^+^-dependent enzymes generate an activated enzyme-nucleotide monophosphate adduct that is inherently the first intermediate of the ligation process. This intermediate contains a phosphoramide bond between the amino group of an active site lysine and the 5′-phosphate of AMP. During the ligation process the 5′-phosphate group of the nucleotide monophosphate is transferred from the amino acid residue to the phosphorylated 5′-end of a DNA where it forms a pyrophosphate linkage. Thus, the 5′-adenosine mono phosphate activates the 5′-phosphate of a DNA substrate for phosphodiester bond formation (Fig. 6A, upper panel)^11^. Consequently, we considered that if the modification carried out by PARP3 on DNA is an ADP-ribose at the 5′-phosphate group of the oligonucleotide, then such a modified DNA strand could potentially serve as a substrate with an activated 5′-phosphate for phosphodiester bond formation by DNA ligase in the absence of ATP (Fig. 6A). In this case the catalysis will be promoted exclusively by a non-activated form of the enzyme, which does not contain AMP-moiety bound to lysine residue. As soon as the DNA ligase accepts ATP in the active site, the reaction is aborted. We tested this hypothesis for various types of DNA ligases using specific DNA duplexes containing the PARP3-modification inside a gap or a nick. The enzymatic list included the widely-used DNA ligase from phage T4, two types of thermophilic DNA ligases, Mth and Pfu, and two human DNA ligases LigI and LigIIIa, which are essential for the various repair processes including the PARP-dependent pathways^12^. The results were the same for all types of DNA ligases. The figure shows an experiment with LigIIIa (Fig. 6B). The set of DNA substrates included gapped and nicked DNA duplexes containing an ADP-ribose residue at the 5′-end of the [^32^P]-labelled oligonucleotide or lacking this residue. To achieve this, we used an annealing of the corresponding modified or unmodified purified oligonucleotides (Fig. 6B,C, lanes 2–16). The details of the purification of the PARP3-modified oligonucleotide are described in the Materials and Methods section. As expected, the full-length product was observed in the absence of ATP when the modified gapped DNA was used (Fig. 6B,C lane 10). The additional evidence giving a consequential argument in support of our theory was obtained in the experiment with apurinic/apyrimidinic endonuclease 1, APE1 (see Supplementary Fig. S1). This enzyme specifically cleaves AP sites during base excision repair process. The experiment presented on the Fig. S1 clearly demonstrates the kinetic mode of cleavage of the ligated product by APE1, which is a circumstantial evidence for the abasic site existence.

However, we also observed a band with an electrophoretic mobility lying between the unmodified and modified oligonucleotides (Fig. 6B,C lane 7, 8, and 10). Based on the ligation mechanism, it seems likely that these bands could arise from an unmodified oligonucleotide with a 5′-pRibp-fragment that is an outcome of the ligase action. It should be noted that the stability of the PARP3-modified DNA differs from that of an ordinary phosphodiester bond that leads to the cleavage of the modified DNA to the initial DNA strand (data not shown). So, we can see the 5–10% of “initial DNA band” on the radio autograph under experiment conditions. In this case, the band with an intermediate electrophoretic mobility is also formed; however, the quantity of the product is low, and it cannot be seen on the autograph. Therefore, the ligation product using modified nicked DNA is formed from the initial DNA strand (Fig. 6B,C lanes 21–23).

### Synthesis of poly(ADP-ribose)polymers by PARP1 and PARP2

**Figure 7:**
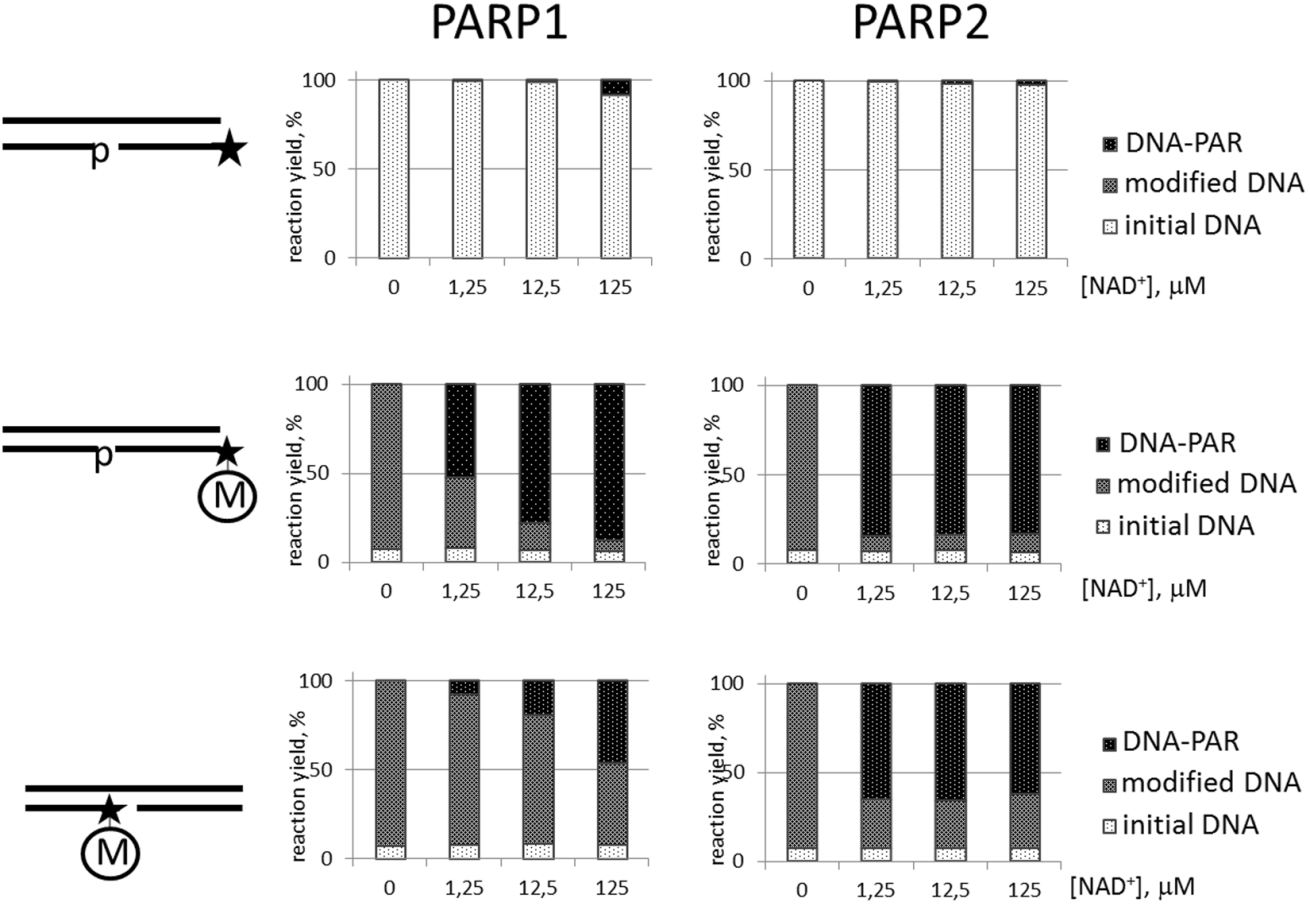
The catalysis of DNA ADP-ribosylation by 0.1 µM purified hPARP1 (left panels) and 0.1 µM mPARP2 (right panels) using various DNA substrates: unmodified gapped DNA at the upper panel, modified gapped DNA with the ADP-ribose residue on the blunt end at the middle panel, and modified gapped DNA with the ADP-ribose residue inside the gap at the bottom panel. The histograms indicate the percentage of processing of the [^32^P]-labelled DNA oligonucleotides. The reactions were performed under increasing concentrations of NAD^+^ (1.25, 12.5 or 125 µM NAD^+^). “M” inside the circle marks the ADP-ribose residue. The asterisk marks the [^32^P]-label.

It was of interest to determine whether the modification to the DNA carried out by PARP3 influenced the subsequent PAR growth catalysed by PARP1 and PARP2. We suggested that transferring ADP-ribose to a 5′-phosphate group of the upstream oligonucleotide could act as a primer for DNA-PARylation by other DNA-dependent PARP members such as PARP1 or PARP2. To address this possibility, we used modified gapped DNAs as substrates in the poly(ADP-ribosyl)ation reactions catalysed by PARP1 and PARP2 (Fig. 7). The results showed that both poly(ADP-ribose)polymerases can exploit the mono(ADP-ribose)-end of DNA to effectively produce long polymers. Moreover, we found a very interesting result concerning to the processivity of PARPs. Specifically, with respect to the PARylation of proteins, PARP1 is more processive than PARP2^13^. In contrast, for the DNA PARylation using primed DNA substrates, PARP2 displayed higher processivity than PARP1.

### Synthesis of poly(ADP-ribose)polymers by HEK cellular extract proteins

**Figure 8:**
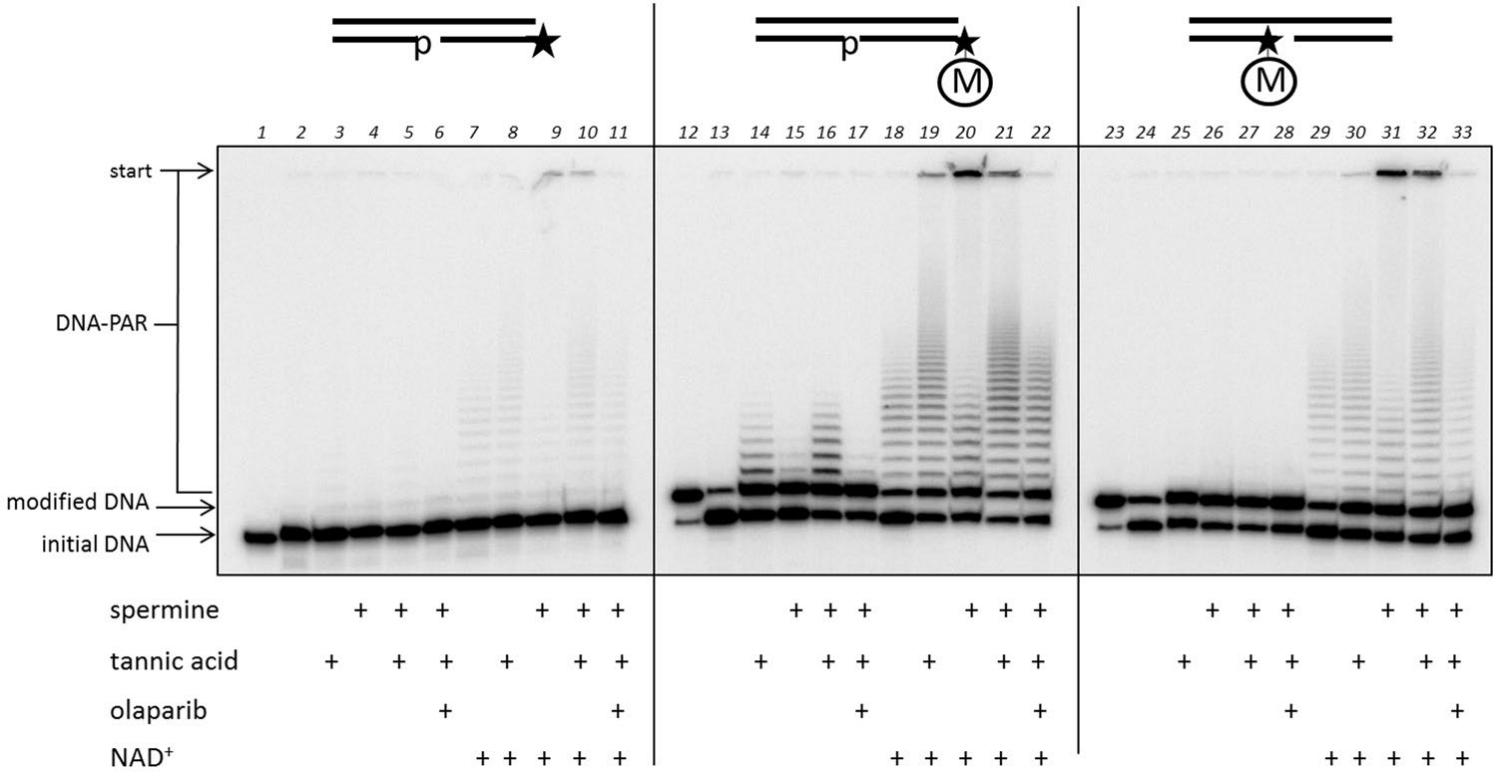
The catalysis of DNA ADP-ribosylation by HEK293 cell-free extract proteins using various DNA substrates in the presence of endogenous (lanes 2–6, 13–17 and 24–28) or 500 µM of added NAD^+^ (lanes 7–11, 18–22 and 29–33). The reactions were performed in buffer with 10 mM EDTA with the addition of spermine, tannic acid or olaparib alone or in combinations. Lanes 1, 12 and 23 – the initial electrophoretic mobility of [^32^P]-labelled oligonucleotides. “M” inside the circle marks the ADP-ribose residue. The asterisk marks the [^32^P]-label.

Since a primed DNA could be utilised by purified proteins as a primary substrate for the growth of a PAR chain on DNA, we investigated this phenomenon as performed by cell-free extract proteins. To test this hypothesis we used cell-free extracts of the HEK293 cell line. It should be noted that these cellular extracts potentially contain all types of enzymes involved in DNA and PAR metabolism. In accordance with this possibility we performed the experiments in the presence of EDTA to avoid DNA degradation and in the presence or absence of spermine, which is a cofactor for DNA-dependent PARPs, of tannic acid, which is an inhibitor of PAR degradation enzymes, and of olaparib, which is a PARP1/2 inhibitor, and examined the differences in PAR synthesis (Fig. 8). It is important to note that the quantity of the DNA and extract proteins were equal in all reaction mixtures. The first unexpected result was that the cellular proteins could synthesis DNA-associated PAR even using a non-primed DNA duplex (Fig. 8, lanes 1–11). Secondly, the PAR synthesis catalysed by the proteins of the HEK extract uses an inherent NAD^+^ resource (Fig. 8, lanes 2–5, 13–17, 24–28). The reaction level, in this case, is not very high compared to that achieved in the presence exogenous NAD^+^, but the pattern of the reaction products was similar and depended primarily on the initial DNA structure. Moreover, the fact that set and quantity of the PAR-DNA adducts were dependent on the NAD^+^ concentration could be considered additional evidence that this activity belongs to PARP proteins (data not shown).

## Discussion

The new member of the PARP family, PARP3, was initially found when searching the expressed sequence tag library of the GenBank database using the sequence of the catalytic domain of hPARP1^14^. Analysis of the human gene revealed the existence of two proteins as alternative splicing variants that differ by 7 aa at the N-terminus^15^. The long splice variant was identified as a core component of the centrosome at all stages of the cell cycle, whereas the short isoform accumulates within the nucleus^15^. During interphase in human somatic cells, PARP3 is primarily localised in cytoplasm and nuclear punctae^16^. Interestingly, overexpression of PARP3 or its N-terminal domain leads to G1/S cell cycle arrest even in DNA-damaged human cells^15^. At the same time, PARP3 is not essential for G2/M cell-cycle checkpoint after ionising radiation^17^.

The use of Northern blotting to study the mRNA expression pattern of mouse PARP3, which is an orthologue of human PARP3, revealed that the mRNA expression level is regulated in a tissue-specific manner and differs remarkably from that of the ubiquitously-expressed PARP1 and PARP2^18^. However, it was later demonstrated that as a protein PARP3 is genuinely expressed at a low level in most tissues of the cynomolgous monkey and that its expression is tightly regulated^19^. Moreover, expression of PARP3 is generally higher in well-differentiated cells such as the neurons of the terminal ganglia and ductal epithelial cells of several secretory tissues than in immature or proliferating cells such as mature lymphocytes or oocytes.

Human PARP3 itself does not contain a specific DNA-binding domain; however, it binds to and is activated by DNA^13,15^. In the absence of DNA, the PARP3 enzymatic autoribosylation activity for is very low and is difficult to discern above background^3^. DNA-dependent PARP3 activity appears to a greater extent following interaction with 5′-phosphorylated DNA breaks^13^. Finally, PARP3 activity is stimulated by DSBs *in vitro*^20^.

At the cellular level, PARP3 accumulates at laser-induced DNA damage sites in human, monkey, and mouse cells, and this accumulation is independent of its activity towards proteins^17^. At the same time, the loss of PARP3 in either human cells or mice did not significantly impact on the long-term survival after irradiation, but PARP3-depleted MRC5 cells displayed spontaneous genome instability that could be one of the consequences of incorrect DSBR^17^. The PARP3 gene is located in the 3p21.3 area of chromosome 3^14^. It is known that this region is altered in several types of malignant tumours^21,22^.

A potential role for PARP3 in cellular processes in response to DNA damage has been suggested on the basis of its interaction with the components of the repair machinery. PARP3 has been reported to co-immunoprecipitate with polycomb protein complexes and it interacts with various of DBS/NHEJ repair proteins such as APLF, Ku-protein and XRCC4/Lig4^20,23^. Additionally, Yasui and his group identified a Ku70/Ku80-dependent recruitment of the human polycomb group member PHF1 to DSB where it contributes to the efficiency of the NHEJ pathway^24^. Furthermore, the ability of the XRCC4/Lig4 complex to overexpress in PARP3-depleted cells suggested that the reducing rates of chromosomal DSB repair obtained in these cells following ionising radiation resulted from inefficient DNA ligation during the NHEJ process^20^. In the meantime, overexpression of APLF led to a restoration of the rate of DSBR even in PARP3-depleted cells. The explanation of these results could be that PARP3 is somehow involved in the existence of APLF-DNA complex and maintenance of its time-of-life, which prolong the XRCC4/Lig4 retention on DNA and subsequent DSB ligation.

Therefore, in all of the cellular events, described above the detailed role of PARP3 is unclear and is mostly attributed to the protein-protein interactions or to protein modifications by PARP3^25^. In the present study, we described a new activity of PARP3 to modify DNA. Biochemical and physical analysis revealed that *in vitro* and in cell-free extracts PARP3 modified the 5′-phosphate of gapped DNA, which resulted from the transfer of ADP-ribose from the NAD^+^-molecule. Based on the collective literature, PARP3 is most probably involved in DSB repair via non-canonical end-joining in its classical variant. The NHEJ process is based on ungoverned ligation of the two DNA termini that had been created as a result of DNA breakage. C-NHEJ can occur throughout the cell cycle but is dominant in the G0/G1 and G2 phases, which correlates well with the expression pattern of PARP3^15,26,27^. The principal scheme for c-NHEJ is relatively simple and comprises a few sequential steps starting from recognition of the DNA ends by the Ku70/80 heterodimer that recruits DNA-PKcs. If necessary, the DNA ends can be trimmed by specific nucleases or filled in by DNA polymerases to create compatible ends before ligation. In some cases, ligation by the XRCC4/Ligase IV complex in combination with APLF and XLF takes place directly without processing of the DNA ends, which leads to complete DNA strands^26^. Taking into account that PARP3 can ADP-ribosylate the 5′-phosphate of the DNA, we can speculate on the possible roles of this DNA modification during DSBR via c-NHEJ. First, such modification could promote retention of the blunt ends of a double-stranded DNA break until the complete repair complex is formed. Thus, DNA-ADP-ribosylation could play a temporary role in saving broken DNA and prolonging the time-of-life of the genome DNA molecule. This proposition is additionally supported by the following observations. First, the DNA modification catalysed by NAD^+^-dependent enzymes is not unique. Surprisingly, a recent study showed that the bacterial cells possess a toxin-antitoxin system DarTG that can ADP-ribosylate DNA at the G-base, and this modification is reversible^28–30^. Second, fairly recently it was published that purified human PARP3 is able to transfer a mono(ADP-ribose) to the double-stranded DNA ends. More importantly, this modification is also reversible by the action of several cellular hydrolases^31^.

Another possible role of mono(ADP-ribosyl)ation is suggested by the existence of two types of ADP-ribosylation. Computational analysis of the amino acid sequences in combination with the biochemical data led to the finding that each member of the PARP family is able to generate either PAR- or MAR-adducts, but not both^32^. However, the existence of both types of modification indicates their important functions in cellular physiology. In our case, for instance, MAR modification could serve as a primer for further elongation to PAR by specific PARP proteins. It is supposed that PAR-chains act as protein binding scaffold that form a specific compartment during DNA-damage stress responses, which require the rapid assembly of a multiprotein complex. The central poly(ADP-ribosyl)polymerase for protein acceptors in the cell is PARP1; nevertheless, PARP2 can also catalyse a long stretch of PAR with reduced processivity^1^. Both PARP1 and PARP2 are required for the recruitment of the nuclease MRE11 in the DSBR process. At the same time, PARP2 depletion leads to an increase in the sensitivity to ionising radiation and alkylating agents, and PARP2^−/−^ cells exhibit slower kinetics of re-joining DNA strand breaks^33^. In our study, we showed that DNA primed by ADP-ribose could be used as a substrate for PAR chain elongation by the purified proteins PARP1 and PARP2 as well as by cell-free extract proteins. More importantly, in contrast to protein PARylation, the PARP2 catalytic activity to extend the ADP-ribose tract attached to DNA was higher than that of PARP1. This observation could indicate the specificity of this reaction and the possible implication of PARP2 in DSB repair processes. This speculation is consistent with *in vitro* data that shown hetero-dimerisation and activation of PARPs with different PAR and MAR activities^3,15,34^.

Finally, we clearly demonstrated here that ADP-ribosylated gapped DNA could be ligated to yield whole dsDNA independently of the type of DNA ligase used. Double-stranded DNA containing an abnormal AP site could serve as substrate for BER in the next stages of DNA repair and to be cleaved by APE1. The consequence of this discovery comes from the following hypothesis. Despite the principal mutagenicity of c-NHEJ, it presents a substantial advantage in rapid kinetics that protects the genome integrity later on^35^. This fast response can be especially important in specific types of cells such as differentiated neurons or epithelial cells, whose DNA cannot be replicated and repaired during the G2 phase through error-free homologous recombination. After DNA damage and the formation of DSB, the immediate quick response to preserve the chromosomal integrity could be more important than accurate but lagging repair. So, first DSBR through c-NHEJ with a ligation of the ADP-ribosylated DNA ends followed by BER with an AP-DNA cleavage by APE1 could be preferable in contrast to error-free but slow homologous recombination.

## Materials and Methods

### Materials

The synthetic oligonucleotides were obtained from Biosset (Novosibirsk, Russia). The reagents for electrophoresis and the basic components of buffers were from Sigma (USA). γ–[^32^P]-ATP and α–[^32^P]-ATP (with specific activities of 5000 and 3000 Ci/mmol, respectively) were from the Laboratory of Biotechnology (Institute of Chemical Biology and Fundamental Medicine, Novosibirsk, Russia). T4 polynucleotide kinase was from Biosan (Novosibirsk, Russia). Ultrapure dNTPs/ddNTPs/NTPs/NAD + were from Promega (USA). SUMO fusion expression vector pETHSUL (GenBank: EF205333.1) and pSUPER vector coding for the catalytic domain of the *S*.*cerevisiae* SUMO hydrolase dtUD1 were kindly provided by Dr. P. Loll (Drexel University, Philadelphia, USA). His_6_ and Strep-II double-tagged dtUD1 hydrolase was expressed and purified as described in ref.^36^.

### Protein purification

Human recombinant PARP3 was purified from *E*. *coli* BL21-CodonPlus(DE3)-RIL cells using a plasmid that encoded the chimeric conjugate of PARP3 with the SUMO-protein and a his-tag on the N-end. The plasmid was made by G. Zarkovich (Gustave Roussy, Université Paris-Saclay, France). The cells were grown on OD (600) of 0.6 at 37 °С Luria Broth with 50 µg/mL ampicillin and 25 µg/mL chloramphenicol. Then they were subsequently dispersed in Terrific Broth medium with 50 µg/mL ampicillin and an additional 12 g/L glycerol. The induction was started by the addition of IPTG to a final concentration of 0.5 mM. The cells were grown at 18 °С for 20 h under constant stirring at 150 rpm to OD (600) of 8.0. They were harvested by centrifugation and resuspended in lysis buffer (50 mМ Na_3_PO_4_, 10 mМ imidazole, 500 mМ NaCl, 10% glycerol, 5 mМ beta-mercaptoethanol, pH 7.5) with a mixture of cOmplete EDTA-free Protease Inhibitors (Roche), followed by treatment with Benzonase and sonication on ice. The supernatant was subjected to chromatography on Ni-chelating resin and eluted with 500 mM imidazole and subsequently applied to a heparin-sepharose resin in 100 mM NaCl. The chimeric protein was removed using a 0.1–1 M NaCl gradient. Hydrolysis of the SUMO fragment was performed at 18 °С for 1 h in a solution that contained the specific hydrolase dtUD1, whereupon the target protein was separated on Ni-chelating and mono-Q resins. Additional fractionation was performed using heparin-sepharose with a 0.1–1 M NaCl gradient. The fractions were collected and dialysed up to 50 mM NaCl and 40% glycerol and stored at −20 °С. The yield was 36 mg from 10.6 g biomass.

PARP1 and PARP2 were purified according to protocols designed earlier^37,38^. T4 DNA ligase was from New England Biolabs (NEB); Pfu DNA ligase was kindly provided by Oskorbin I, ICBFM, Novosibirsk; Mth DNA ligase and human APE1 was kindly provided by Khodyreva SN, ICBFM, Novosibirsk; human DNA ligase I was kindly provided by Dyrkheeva NS, ICBFM, Novosibirsk; human DNA ligase III was kindly provided by Vasil’eva IA, ICBFM, Novosibirsk; poly(ADP-ribose)glycohydrolase (PARG) was kindly provided by Kutuzov MM and Ilina ES, ICBFM, Novosibirsk.

### HEK293 cellular extract

The growth of HEK293 cells and the preparation of the cell-free extracts were performed by E.S. Ilina, ICBFM, Novosibirsk. Specifically, HEK293 cells were cultured in IMDM to reach 80% confluence, then washed three times with an ice-cold phosphate-buffered saline and resuspended at 10^6^ cells/20 μl in buffer I (10 mM Tris-HCl, pH 7.8, and 200 mM NaCl, and cOmplete Protease Inhibitor Cocktail (Roche) at the recommended concentration). After the addition of an equal volume of buffer II (10 mM Tris-Cl, pH 7.8, 200 mM NaCl, 2 mM EDTA, 40% glycerol, 0.2% Nonidet P-40, 2 mM dithiothreitol, cOmplete Protease Inhibitor Cocktail at the recommended concentration) the cell suspension was rocked at 4 °C for 1 h and then centrifuged at 16,000xg for 10 min. The supernatant was recovered and stored in small aliquots at −80 °C.

### Oligonucleotide substrates

The oligodeoxyribonucleotides were 5′-[^32^P]-phosphorylated with T4 polynucleotide kinase as described^39^. Unreacted γ–[^32^P]-ATP was removed by passing the mixture over a MicroSpinTM G-25 column (Amersham Pharmacia Biotech, GE Healthcare, USA) using the manufacturer’s protocol. The labelled oligonucleotides were precipitated from the solution by 4% LiClO_4_ in acetone and dissolved to at the necessary concentration in water. Complementary oligodeoxynucleotides were annealed in equimolar amounts by heating a water solution to 95 °C for 10 min or where indicated up to 80 °C for 2 min, followed by slow cooling to room temperature with the stops at specific temperatures according to the melting temperature of the corresponding oligonucleotides. The following oligonucleotide sequences were used for the construction of DNA duplexes: whole strands 5′-(d)GGCTTCATCGTTGTCTCAGACCTGGTGGATACCG−3′ and 5′-(d)CAGACCTGGTGGATACCG*TCTCATTTCATCTGGCTTGCG*-3′ with or without 5′-phosphate groups; upstream oligonucleotides 5′-(d)CGGTATCCACCAGGTCTG-3′, 5′-(d)GACAACGATGAAGCC-3′ with or without 5′-phosphate groups; downstream oligonucleotides 5′-(d)AGACAACGATGAAGCC-3′, 5′-(d)ACAACGATGAAGCC-3′, 5′-(d)ACGATGAAGCC-3′ with or without 5′-phosphate groups; upstream oligonucleotides for ligation assays 5′-(d)*GAGTAAAGTAGACCGAACGC*-3′, 5′-(d)*AGAGTAAAGTAGACCGAACGC*-3′.

### Synthesis of ^32^P-NAD^+^

The synthesis of radioactive NAD^+^ was carried out from α–[^32^P]-ATP. The reaction mixture containing 1 mM ATP, 10 MBq of α–[^32^P]-ATP, 20 mM MgCl_2_, 2 mM beta-nicotinamide mononucleotide, and 5 mg/mL nicotinamide nucleotide adenylyltransferase 1 in 25 mM Tris-HCl, pH 7.5, was incubated at 37 °C for 60 min, then stopped by heating to 80 °C for 5 min. After removal of the denatured protein by centrifugation the solution was used as the reactant.

### Condition screening

The condition selection was performed in the presence of 0–10 mM MgCl_2_ or spermine as a cofactor in HDB buffer solution containing HEPES-KOH, pH 8.6, 0.25 mg/mL BSA and 0.5 mM DTT. Additional experiments were carried out in the presence of 0–10 mM EDTA. The reaction mixtures (final volume 10 µL) contained 0.02 µM [^32^P]-DNA substrate, 0.01, 0.05, 0.1, 0.5 or 1 µM PARP3 and various concentrations of NAD^+^: 1–1000 µM. The reaction mixtures were incubated for 30 min at 37 °С then terminated by adding the 5x gel loading solution (90% formamide, 0.1% xylenecyanole and 0.1% bromophenol blue) and heating at 85 °C for 2 min. The mixtures were resolved on a 20% polyacrylamide gel containing 7 M urea and 10% formamide in 1x TBE buffer. The gels were dried and subjected to autoradiography and/or phosphorimaging for quantitation using the Typhoon imaging system from GE Healthcare Life Sciences and analysed using OriginPro7.5, Microcal Software, USA.

### Purification of PARP3-modified oligonucleotides

The reaction was carried out in 400 µL HDB buffer with 2 mM MgCl_2_. The mixture containing 1 µM [^32^P]-DNA substrate gap1, 5 µM PARP3 and 1 mM NAD^+^ was incubated at 37 °C for 45 min, then heated at 80 °C for 5 min and loaded onto a 20% polyacrylamide gel containing 7 M urea and 10% formamide in TBE buffer with 100 µL of 5x loading solution. After separation, the reaction products were subjected to autoradiography, and the corresponding band was extracted twice in 1 mL of 150 mM NaCl by passive elution at 4 °С for a few hours. The target oligonucleotide was precipitated from the solution by 4% LiClO_4_ in acetone and dissolved at the necessary concentration in water. The complementary oligodeoxynucleotides were annealed in equimolar amounts by heating a water solution to 85 °C for 2 min, followed by slow cooling to room temperature with stops at specific temperatures according to the melting temperature of the corresponding oligonucleotides.

### Degradation of PARP3 modification

The various types of modified DNA were processed using PARG or Nudix enzymes. For this, the PARP3-modified DNA substrates prepared as described above and the PARP2-modified DNA substrate prepared *in situ* were used. During the *in situ* experiment the reaction mixtures (final volume 10 µL), which contained 0.02 µM [^32^P]-DNA, 0.1 µM PARP2 and 1 mM NAD^+^ in HDB buffer with 2 mM MgCl_2_, were incubated for 30 min at 37 °С. After this procedure, the DNAs were used as follows. Reaction mixtures with 0.02 µM of the various types of DNA were incubated with 0.25 µM PARG in HDB buffer for 30 min at 37 °С or 10 µM Nudix in HDB buffer with 10 mM MgCl_2_ for 20 h at 37 °С. The reactions were terminated and analysed as described above.

### Identification of PARP3 modification

The reaction was carried out in 3 mL of HDB buffer with 2 mM MgCl_2_. The mixture, which contained 1 µM DNA substrate gap1, 5 µM PARP3 and 1 mM NAD^+^, was incubated at 37 °C for 45 min, after which it was heated at 80 °C for 5 min and loaded onto a 150 µL DEAE-toyoperl (Sigma-Aldrich, USA), at 4 °C for 8 h. After washing 3 times with 300 µL 10 mM Tris-HCl, pH 8.0, the DNA was eluted with 3 aliquots of 60 µL of 1 M LiClO_4_, precipitated with 4% LiClO_4_ in acetone, and dissolved in 40 µL of water and 20 µL of 5x loading solution. The target oligonucleotide was purified using denaturing PAGE as described above, dissolved in 10 µL of water and subjected to MALDI-TOF analysis on a REFLEX III instrument (Bruker Daltonics, Germany).

### The influence of the 3′-end motif of the primer on PARP3 activity

To discriminate the type of motif that is important for PARP3 action, partial DNA duplexes with the various 3′-moieties in the priming oligonucleotide were constructed *in situ*. The reaction mixtures (final volume 10 µL) containing 0.02 µM [^32^P]-DNA substrate gap1, 0.1 µM DNA polymerase beta and 25 µM ATP µM, 10 µM ddATP or 10 µM dATP in HDB buffer with 2 mM MgCl_2_ were incubated for 30 min at 37 °С. The modified [^32^P]-DNA substrates were used to investigate PARP3 activity. For this, 0.1 µM PARP3 and 3, 20, 100 or 500 µM NAD^+^ were added to the DNA mixture. The reactions were analysed as described above after incubation at 37 °C for 30 min.

### The influence of the 5′-end motifs on PARP3 activity

Partial DNA duplexes with a 5′-phosphate or 5′-hydroxide group on the different parts of the DNA strands were constructed by annealing equimolar amounts of the corresponding oligonucleotides. In some cases, the DNA substrates contained their own [^32^P]-label, in other cases the [^32^P]-label was introduced by PARP3 from [^32^P]-NAD^+^ during the experiment. The reaction mixtures (final volume 10 µL) containing 0.02 µM DNA substrate, 0.1 µM PARP3 and 5, 50 or 500 µM NAD + alone or in combination with 5 µM [^32^P]-NAD^+^ in HDB buffer with 2 mM MgCl_2_ were incubated for 30 min at 37 °С. The reactions were terminated and analysed as described above.

### Ligation assay

The reactions were performed using various enzymes: T4 DNA ligase, Pfu DNA ligase, Mth DNA ligase, human DNA ligase I, or human DNA ligase IIIa. At the first step, DNA substrates containing 5′-ADP-ribose inside the gap or nick structures were obtained by annealing equimolar amounts of the corresponding oligonucleotides. The reaction mixtures (final volume 10 µL) containing 0.02 µM [^32^P]-DNA substrate, 0.1 µM DNA ligase and, if indicated, 0.2 mM or 1 mM ATP in HDB buffer with 10 mM MgCl_2_ were incubated for 30 min in various temperature conditions: for T4 DNA ligase at 24 °С, for DNA ligase I and DNA ligase IIIa at 37 °С, for Mth DNA ligase at 50 °С, and for Pfu DNA ligase at 60 °С. All probes were analysed as described above.

### Synthesis of poly(ADP-ribose)polymers by PARP1 and PARP2

Partial DNA duplexes with or without ADP-ribose in [^32^P]-labelled oligonucleotides were constructed by annealing equimolar amounts of the corresponding oligonucleotides. The reaction mixtures (final volume 10 µL) containing 0.02 µM DNA substrate, 0.05, 0.1 or 0.5 µM PARP1 or PARP2 and 5, 50 or 500 µM NAD^+^ in HDB buffer with 2 mM MgCl_2_ were incubated for 20 min at 37 °С. The reactions were terminated and analysed as described above.

### Synthesis of poly(ADP-ribose)polymers by HEK cellular extract proteins

Partial DNA duplexes with or without ADP-ribose in the [^32^P]-labelled oligonucleotides were constructed by annealing equimolar amounts of the corresponding oligonucleotides. The reaction mixtures (final volume 20 µL) containing 0.05 µM DNA substrate and 0.6 mg/mL HEK extract only or with the addition of 500 µM NAD^+^ in HDB buffer with 10 mM EDTA, pH 8.6, and 100 mM NaCl were incubated for 20 min at 37 °С. Additional reaction mixtures contained 100 µM tannic acid and/or 2 mM spermine, or 100 µM tannic acid, 2 mM spermine and 1 µM olaparib. The reactions were terminated and analysed as described above.

All data generated or analysed during this study are included in this published article.

## Electronic supplementary material

Supplementary Figure
