# Supplementary material for: Dna is a New Target of Parp3
Source: Sci Rep. 2018 Mar 8;8:4176. doi: 10.1038/s41598-018-22673-3 (PMC5843604; doi:10.1038/s41598-018-22673-3)
Supplement: Supplementary file 1 — Supplementary Figure [file 41598_2018_22673_MOESM1_ESM.pdf]

DNA IS A NEW TARGET OF PARP3

E.A. Belousova[1], A.A. Ishchenko[2,3], O.I. Lavrik[1,4*]

[1] Institute of Chemical Biology and Fundamental Medicine (ICBFM), SB RAS, Lavrentiev Av. 8, Novosibirsk 630090, Russia

[2]Groupe «Réparation de l'ADN», Equipe Labellisée par la Ligue Nationale Contre le Cancer, CNRS UMR8200, Univ. Paris-Sud, Université Paris-Saclay, F-94805 Villejuif, France

[3] Gustave Roussy, Université Paris-Saclay, F-94805 Villejuif, France

[4] Novosibirsk State University, Pirogov Str. 2, Novosibirsk 630090, Russia

*To whom correspondence should be addressed. Tel.: +73833635195; e-mail: lavrik@niboch.nsc.ru

Figure S1. The cleavage of the ligated product by APE1. A) Ligation by DNA ligase IIIa with the followed cleavage by APE1 of the initial one-window gapped (lines 4-8) or nicked (lanes 11-15) DNA substrates. The reactions were performed using consequential addition of DNA ligase IIIa and APE1. At the first step, 0.02 μM [^{32}P]-DNA substrate in HDB buffer with 10 mM $MgCl_2$ were treated by 0.1 μM DNA ligase IIIa for 30 min at 37°C, and an aliquot was selected (lanes 4 and 11). Then the mixtures were treated with 1 nM human APE1 at 37°C for 15, 30, 60 and 600 sec. Subsequently, an aliquot of all samples were selected at each time-point. In control experiments 0.02 μM [^{32}P]-DNA substrate in HDB buffer with 10 mM $MgCl_2$ was treated by 1 nM APE1 for 600 sec at 37°C or 10 μM Nudix for 20 h at 37°C. All mixtures were resolved on a 20% polyacrylamide gel containing 7 M urea and 10% formamide in 1x TBE buffer. The gels were dried and subjected to autoradiography and/or phosphorimaging for quantitation using the Typhoon imaging system from GE Healthcare Life Sciences and analysed using OriginPro7.5, Microcal Software, USA. Here, lane 1 shows the DNA electrophoretic mobility of 18 nt oligonucleotide; lanes 2 show the initial mobility of [^{32}P]-labelled ADP-DNA substrate. Lanes 5-8 and 12-15 represented the kinetic assay for APE1. Lanes 3 and 10 contained corresponding DNA substrates treated by APE1. Substrates gap-mod and nick-mod represent one-window gapped or nicked DNA duplexes assembled by the same whole DNA strand and contained the ADP-ribose residue at the 5'-end of the [^{32}P]-labelled oligonucleotide. "M" inside the circle marks the modification induced by PARP3. The asterisk marks the [^{32}P]-label. (B) Quantitative analysis of the assay reactions. The bars in the charts correspond to the respective lanes on the upper autoradiogram.

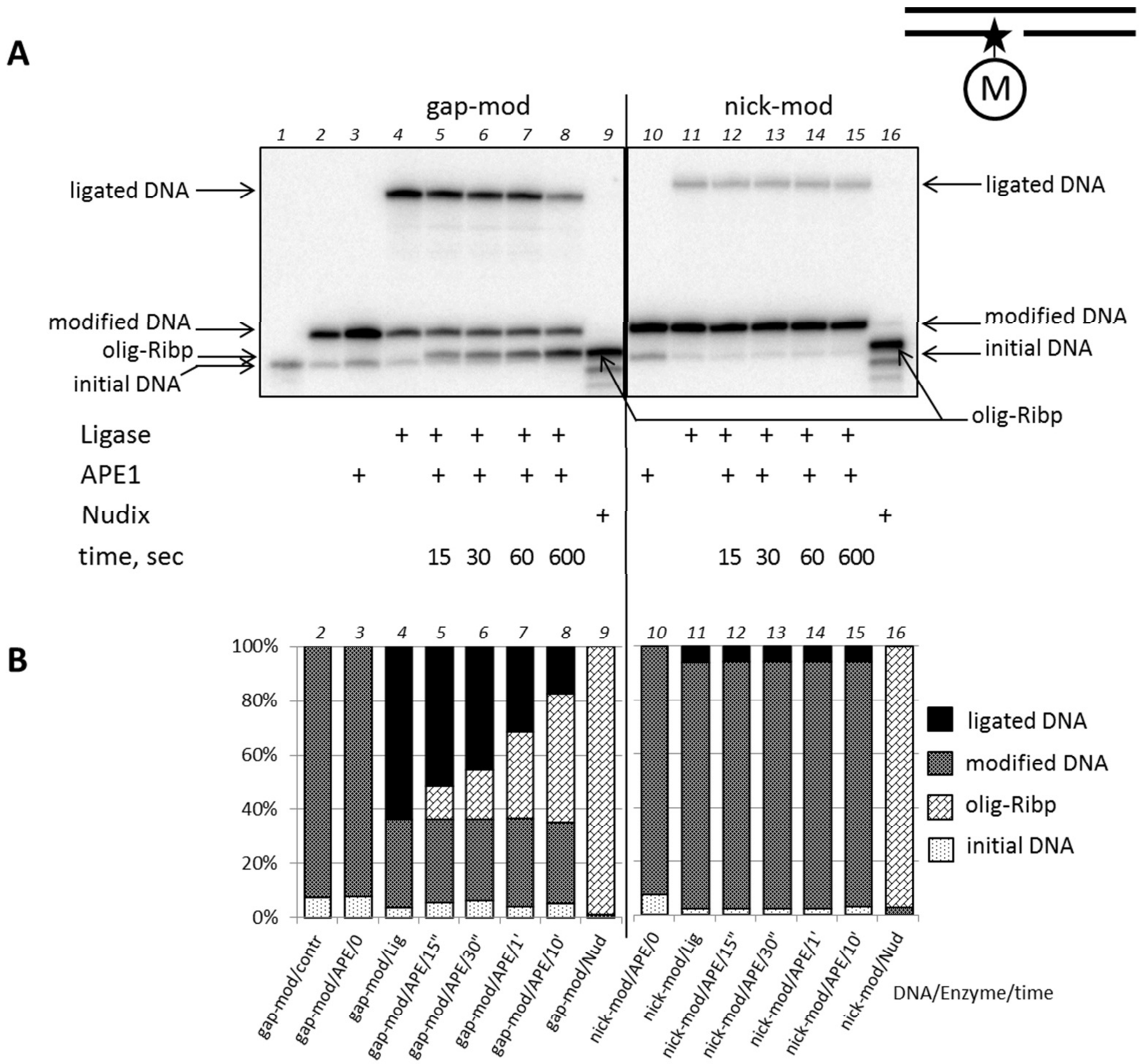
A
B
gap-mod
nick-mod
M
1 2 3 4 5 6 7 8 9 10 11 12 13 14 15 16
ligated DNA
modified DNA
olig-Ribp
initial DNA
Ligase
APE1
Nudix
time, sec
15 30 60 600
100%
80%
60%
40%
20%
0%
gap-mod/contr
gap-mod/APE/0
gap-mod/Lig
gap-mod/APE/15''
gap-mod/APE/30''
gap-mod/APE/1'
gap-mod/APE/10'
gap-mod/Nud
nick-mod/APE/0
nick-mod/Lig
nick-mod/APE/15''
nick-mod/APE/30''
nick-mod/APE/1'
nick-mod/APE/10'
nick-mod/Nud
DNA/Enzyme/time
